# By inhibiting Ras/Raf/ERK and MMP-9, knockdown of EpCAM inhibits breast cancer cell growth and metastasis

**DOI:** 10.18632/oncotarget.4551

**Published:** 2015-09-03

**Authors:** Jiujiao Gao, Xue Liu, Fan Yang, Tingjiao Liu, Qiu Yan, Xuesong Yang

**Affiliations:** ^1^ Department of Biochemistry and Molecular Biology, Dalian Medical University, Liaoning Provincial Core Lab of Glycobiology and Glycoengineering, Dalian, People's Republic of China

**Keywords:** EpCAM, breast cancer, metastasis, MMP-9, Ras/Raf/ERK

## Abstract

Epithelial cell adhesion molecule (EpCAM) is a type I transmembrane protein that is expressed in the majority of normal epithelial tissues and is overexpressed in most epithelial cancers including breast cancer, where it plays an important role in cancer progression. However, the mechanism by which EpCAM promotes the progression of breast cancer is not understood. In this study, we found that EpCAM expression was increased in tumor tissue from breast cancer patients compared to healthy patients. Overexpression of EpCAM in breast cancer cells enhanced tumor cell growth *in vitro* and increased invasiveness, whereas small interfering RNA-mediated silencing of EpCAM (si-EpCAM) had the opposite effect. EpCAM knockdown led to decreased phosphorylation of Raf and ERK, suppression of malignant behavior of breast cancer cells, and inhibition of the Ras/Raf/ERK signaling pathway. Furthermore, si-EpCAM-mediated invasion and metastasis of breast carcinoma cells required the downregulation of matrix metalloproteinase-9 (MMP-9) through inhibition of this signaling pathway. In conclusion, our data show that knockdown of EpCAM can inhibition breast cancer cell growth and metastasis via inhibition of the Ras/Raf/ERK signaling pathway and MMP-9.

## INTRODUCTION

Breast cancer is a malignant tumor that originates from healthy mammary gland cells, and is most frequent among women aged between 50 and 70 years [1]. Nearly 39,510 women were estimated to die in 2013 as a result of breast cancer, accounting for 14% of all cancer-related deaths [2]. Despite the advances that have been made in the treatment of breast cancer, the major cause of death from breast cancer continues to be metastasis.

Epithelial cell adhesion molecule (EpCAM) is a glycosylated, type I transmembrane protein, which is overexpressed in various neoplasms such as breast cancer [3], hepatocellular carcinoma (HCC) [4], glioma [5], and colorectal cancer [6], and is used as a diagnostic and prognostic marker. Increasing clinical evidence has confirmed that EpCAM is involved in cancer progression and is associated with a poor prognosis. For example, the overexpression of EpCAM has been associated with a poor prognosis in patients with pancreatic cancer and HCC [7, 8]. In addition, emerging evidence suggests that EpCAM may be a critical factor in tumor development, progression, and metastasis [9–11]. Recent studies have also revealed that EpCAM regulated miR-181c and miR-130b may play significant roles in retinoblastoma progression [12]. Another report demonstrated the positive roles of EpCAM in the regulation of cell growth and the self-renewal of human keratinocytes [13].

Together, these results strongly suggest a significant role for EpCAM in human cancer progression. However, the role of EpCAM in tumor growth and the mechanism by which EpCAM promotes the metastasis of breast cancer are currently unknown. In this study, we evaluated the effect of changes in EpCAM expression levels on the growth and metastasis of breast cancer cells *in vitro* and *in vivo* through RNA interference or overexpression method. We also explored the role of EpCAM in the progression of breast cancer.

## RESULTS

### Expression of EpCAM was upregulated in breast cancer tissues

**Figure 1:**
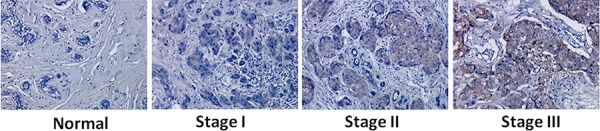
Increased expression of EpCAM in breast cancer and correlation with tumor stage and progression Immunohistochemical staining was performed using an antibody against EpCAM. Representative images of immunohistochemical staining of EpCAM in different breast cancer samples.

EpCAM is overexpressed in breast cancer [14]. To further confirm this relationship, we analyzed EpCAM expression in normal human breast and breast cancer tissue by immunohistochemistry. The expression of EpCAM was evaluated in 120 cases. Basic demographic information for the 120 evaluable cases is presented in Table 1. The mean age at diagnosis was 49.03 years (range 28–74 years). In addition, 76.7% (92/120) of breast cancer specimens were positively stained with anti-EpCAM, according to our criteria (see Materials and Methods). EpCAM expression was confined to the membrane of breast cancer cells in all cases (Fig. 1). Consistent with previous studies, EpCAM expression was significantly associated with tumor stage and tumor grade [15, 16]. In contrast, little or no EpCAM expression was observed in normal breast tissue. EpCAM expression in early-stage (I, II) and advanced-stage (III) breast cancer tissues was significantly higher than that in normal breast tissues. In addition, EpCAM expression in advanced-stage (III) breast cancer tissue was significantly higher than that in early-stage tissue (I, II) (Fig. 1). Higher EpCAM expression was correlated with stage III cancer, but not with stage I or II disease. EpCAM expression was higher in estrogen receptor-negative (ER-) cases (84.6% in ER- cancers versus 74.5% in ER+ cancers, *P* < 0.0001; Table 1) and human epidermal growth factor receptor 2-positive (HER2+) cases (82.5% in HER2+ cancers versus 74.6% in HER2- cancers, *P* < 0.05; Table 1). We also found that higher EpCAM expression was correlated with high Ki67 expression and low p53 expression. It is well known that p53 is the tumor suppressor and has many mechanisms of anticancer function, and plays a role in apoptosis, genomic stability, and inhibition of angiogenesis [17, 18]. Ki-67 is a tumor marker that is found in growing, dividing cells. A high index of Ki-67 usually means a poor prognosis [19]. While EpCAM was high expressed in the cancer cells and was considered as oncogene. Through the results of table 1, we demonstrate that EpCAM was tumor-associated molecular and associated with the progression of breast cancer.

**Table 1:** Association between EpCAM expression and clinicopathological parameters

clinicopathological parameters	EpCAM positive		EpCAM negative		*P* value
Mean age at diagnosis (years)	49.03		47.32		0.654
**Tumor grade**					
I	4	40.0%	6	60.0%	
II	64	74.4%	22	25.6%	
III	24	100%	0	0%	
**Oestrogen receptor**					<0.001
ER+	70	74.5%	24	25.5%	
ER-	22	84.6%	4	15.4%	
**HER2**					<0.05
HER2+	45	82.5%	12	17.5%	
HER2−	47	74.6%	16	25.4%	
**Ki67**					<0.001
Ki67+	80	80.0%	20	20.0%	
Ki67−	12	60.0%	8	40.0%	
**P53**					<0.001
P53+	12	54.5%	10	45.5%	
P53−	80	81.6%	18	18.4%	

Abbreviation: EpCAM = epithelial cell adhesion molecule; ER = oestrogen receptor; HER2 = human epidermal growth factor receptor

### EpCAM is involved in breast cancer proliferation, migration, and invasion *in vitro*

**Figure 2:**
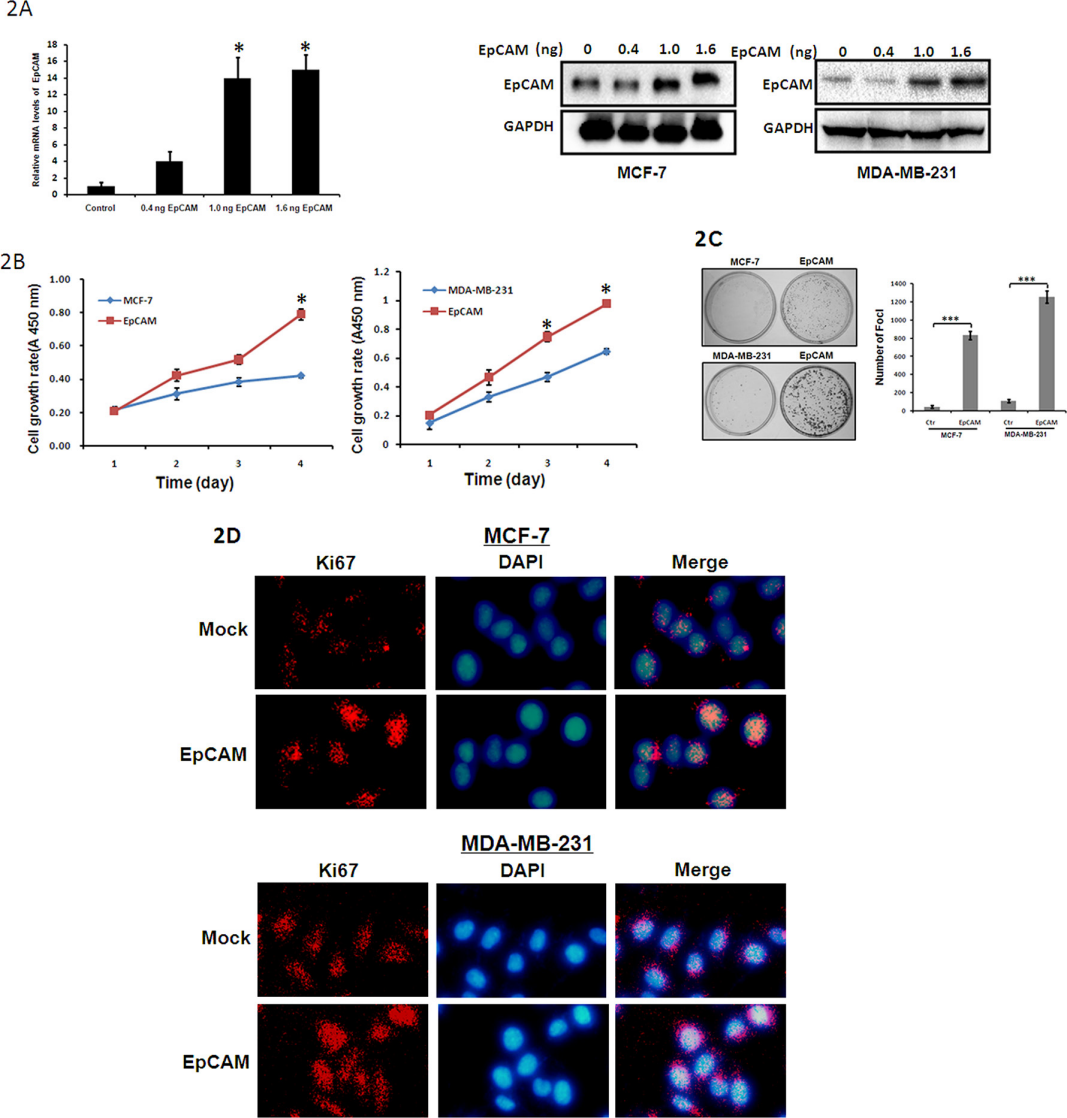
The effect of EpCAM on breast cancer cell metastasis **A.** Cells were transfected with EpCAM expression plasmid (0.4ng, 1.0ng, 1.6ng respectively). Expression of EpCAM was confirmed by qRT–PCR (only in MCF-7 cells) and Western blot analysis. Empty vector-transfected cells were used as controls. **P < 0.01. **B.** Growth curves of EpCAM-transfected cells were compared to control cells with the CCK-8 assay. Points, mean of at least three independent experiments; bars, standard deviation. *P < 0.01. **C.** Representative inhibition of foci formation in monolayer culture by EpCAM, and quantitative analyses of foci numbers. Columns, mean of at least three independent experiments; bars, standard deviation. ***P < 0.001. **D.** Effects of EpCAM on Ki67 in breast cancer cell lines. Ki67 expression was detected by immunofluorescence staining in MCF-7 and MDA-MB-231 cells treated with EpCAM plasmid transfection. Red fluorescence: Ki67; DAPI staining for nuclear DNA.

To elucidate the role of EpCAM in breast cancer progression, an EpCAM overexpression plasmid and small interfering RNA-mediated silencing of EpCAM (si-EpCAM) were used to increase and reduce EpCAM expression in MCF-7 and MDA-MB-231 breast cancer cell lines, respectively. MCF-7 and MDA-MB-231 cells that had been transfected with the EpCAM expression plasmid displayed significantly increased EpCAM expression at both the mRNA and protein levels compared to cells transfected with vector alone (Fig. 2A).

We first explored the effects of EpCAM overexpression on cell growth using the Cell Counting Kit-8 (CCK-8) assay. As shown in Fig. 2B, EpCAM overexpression significantly enhanced the growth of MCF-7 and MDA-MB-231 cells. Next, we performed a clonogenic assay to confirm the effects of EpCAM on proliferation. We found that EpCAM overexpression dramatically increased the colony formation efficiency of MCF-7 and MDA-MB-231 cells (Fig. 2C). Because Ki67 antigen is an important marker of cell proliferation, we next examined Ki67 expression by immunofluorescence staining. As shown in Fig. 2D, we found that the overexpression of EpCAM in MCF-7 and MDA-MB-231 cells significantly upregulated Ki67 staining.

**Figure 3:**
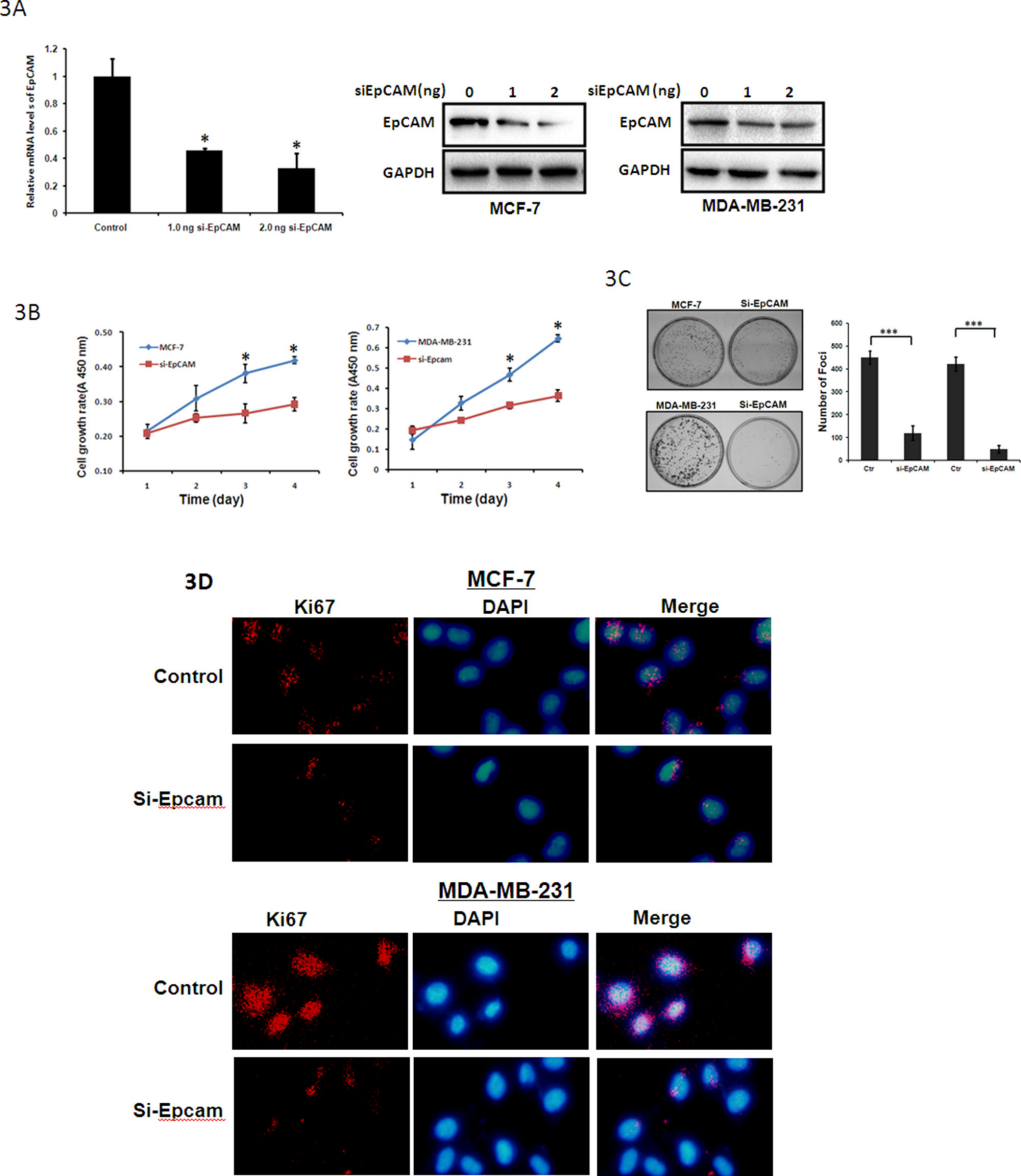
Tumor suppressive functions of si-EpCAM in breast cancer cells **A.** MCF-7 and MDA-MB-231 cells were treated with control siRNA or siRNA against EpCAM (0 ng, 1.0 ng, 2.0 ng respectively). qRT-PCR (only in MCF-7 cells) and Western blot analysis were performed to detect EpCAM expression. GAPDH was used as a loading control. *P < 0.01. **B.** Cell growth rates were determined with a CCK-8 proliferation assay. *P < 0.01. **C.** Representative increase of foci formation in monolayer culture by si-EpCAM and quantitative analyses of foci numbers. Columns, mean of at least three independent experiments; bars, standard deviation. ***P < 0.001. **D.** Effects of si-EpCAM on Ki67 in breast cancer cell lines. Ki67 expression was detected by immunofluorescence staining in MCF-7 and MDA-MB-231 cells treated with si-EpCAM transfection. Red fluorescence: Ki67; DAPI staining for nuclear DNA.

We used si-EpCAM to reduce EpCAM expression. These two cell lines that were transfected with si-EpCAM displayed significantly decreased EpCAM expression at both the mRNA and protein levels compared to control cells (Fig. 3A). EpCAM knockdown decreased the growth of MCF-7 and MDA-MB-231 cells (Fig. 3B). Colony formation efficiency was deduced in si-EpCAM-transfected MCF-7 and MDA-MB-231 cells (Fig. 3C). In addition, knockdown of EpCAM in MCF-7 and MDA-MB-231 cells dramatically downregulated Ki67 expression (Fig. 3D). These results suggest that EpCAM can significantly promote the proliferation of breast cancer cells.

### Knockdown of EpCAM inhibits breast cancer cell mobility

**Figure 4:**
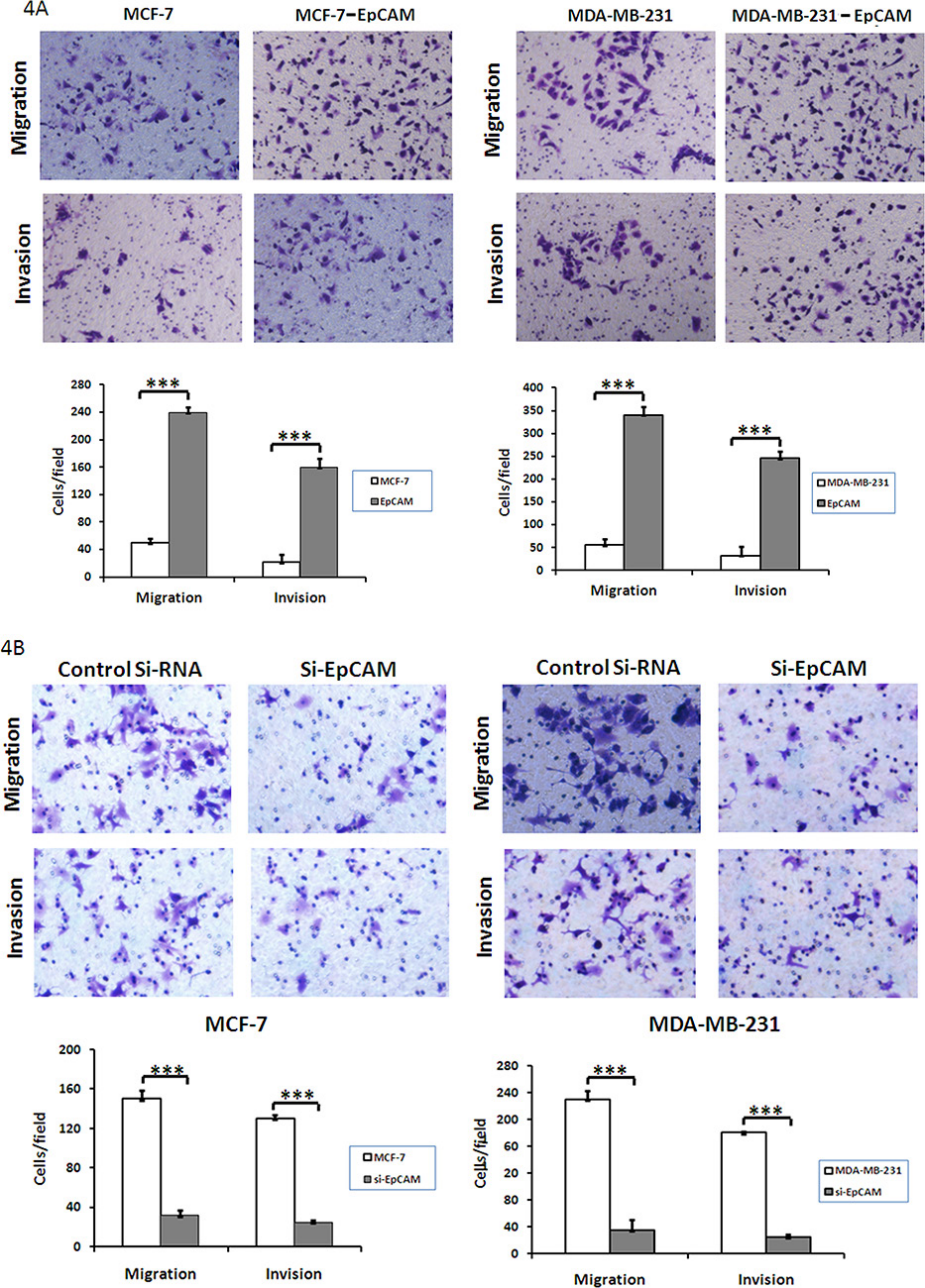
Effect of EpCAM on breast cancer cell migration and invasion **A.** Transwell cell migration and invasion assays were used to compare cell migration and invasion between EpCAM- and vector-transfected cells. The cells that migrated through the polyethylene terephthalate membrane or invaded through the Matrigel were fixed and stained with crystal violet (original magnification 200 ×). The results were expressed as mean ± SD of three independent experiments. ***P < 0.001. The summary graphs are presented for the experiment. **B.** Transwell cell migration and invasion assays were used to compare cell migration and invasion between EpCAM-transfected cells treated with control siRNA or siRNA against EpCAM. The results were expressed as mean ± SD of three independent experiments. ***P < 0.001. The summary graphs are presented for the experiment.

We next used a transwell assay to determine whether EpCAM could affect the ability of breast cancer cells to migrate and invade. EpCAM overexpression promoted both migration (235.46 ± 10.25 versus 47.38 ± 5.48, 339.25 ± 18.35 versus 55.72 ± 16.56; *P* < 0.0001) and invasion (160.64 ± 15.28 versus 22.69 ± 8.72, 245.67 ± 15.67 versus 32.64 ± 14.94; *P* < 0.0001) in MCF-7 and MDA-MB-231 cells, respectively (Fig. 4A). In addition, EpCAM knockdown significantly inhibited cell migration (150.13 ± 8.74 versus 32.32 ± 5.78, 230.58 ± 13.46 versus 35.16 ± 2.27; *P* < 0.001) and invasion (130.61 ± 5.27 versus 25.49 ± 2.47, 180.19 ± 15.60 versus 26.31 ± 3.49; *P* < 0.001) in MCF-7 and MDA-MB-231 cells (Fig. 4B). These results indicate that EpCAM significantly affects the invasion and migration of breast cancer cells.

### EpCAM promotes cell growth and invasion via the Ras/Raf/ERK signaling pathway and MMP-9

**Figure 5:**
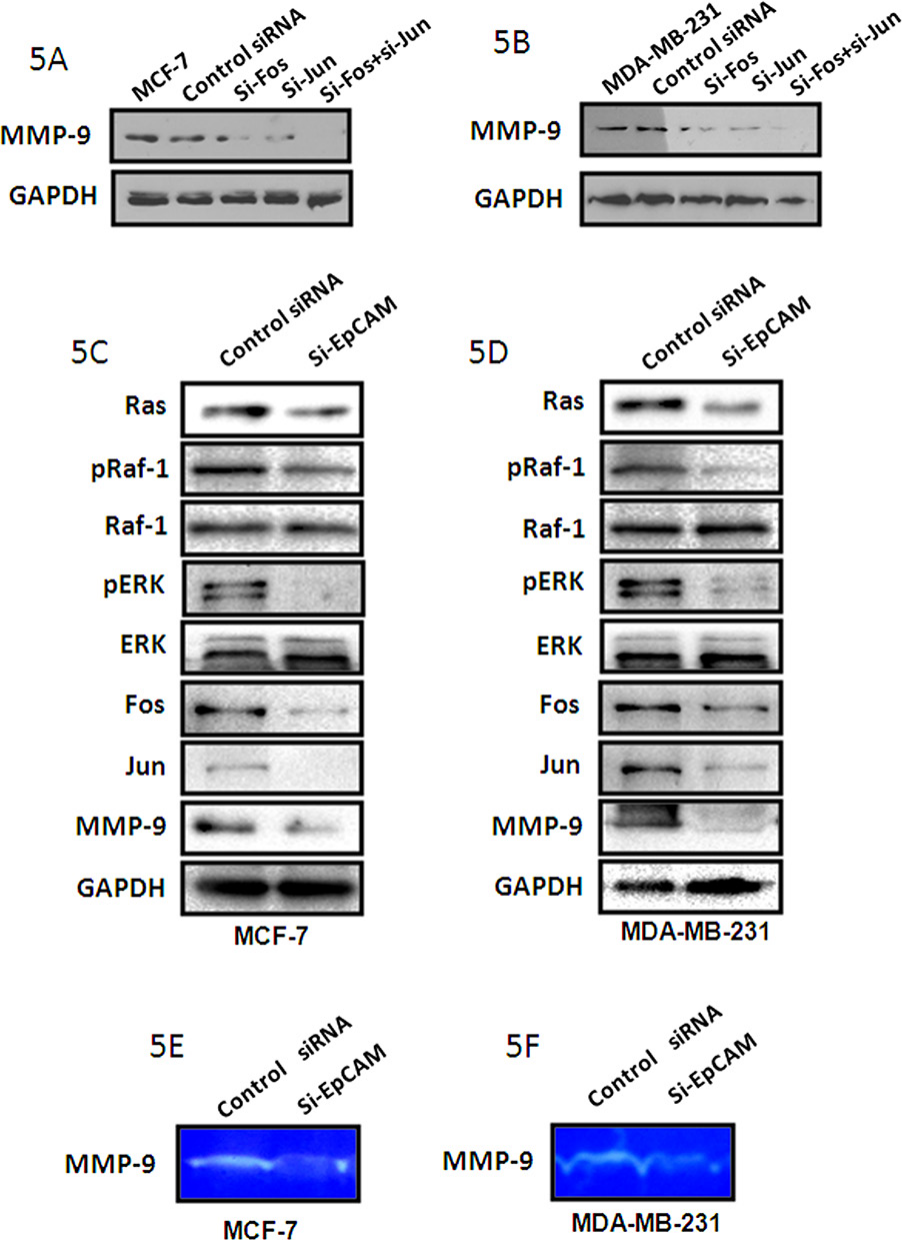
si-EpCAM inhibited cell growth and invasion via the Ras/Raf/ERK pathway and MMP-9 **A, B.** MCF-7 and MDA-MB-231 cells were treated with si-Fos and/or si-Jun, respectively. MMP-9 expression was detected with the method of Western blot. GAPDH was used as a loading control. **C, D.** EpCAM knockdown by siRNA markedly inhibited phosphorylation of Raf and ERK, and downregulated the level of MMP-9 through Fos and Jun in the MCF-7 and MDA-MB-231 cells. Expression levels of these proteins were assessed by immunoblotting; GAPDH was used as a loading control. **E, F.** MMP9 activities were determined using the gelatin-based zymography assay.

Some papers have demonstrated that Fos and Jun are transcription factors that bind to the MMP-9 promoter region [20, 21]. We have demonstrated that MMP-9 expression decreased when knockdown Fos and/or Jun with the si-RNA sequences in both MCF-7 cells and MDA-MB-231 cells (Fig. 5A, 5B). To assess the effect of EpCAM expression on Raf and ERK activities, we examined the phosphorylation of Raf. EpCAM knockdown markedly suppressed Raf phosphorylation in MCF-7 and MDA-MB-231 cells (Fig. 5C, 5D). We also evaluated the molecular consequence of EpCAM knockdown by examining components of the Ras/Raf/ERK signaling pathway in these two cell lines. The results showed that si-EpCAM decreased the expression of Ras, pRaf, and pERK. Together, these data indicate that EpCAM knockdown markedly inhibits the Ras/Raf/ERK signaling pathway in MCF-7 and MDA-MB-231 cells.

**Figure 6:**
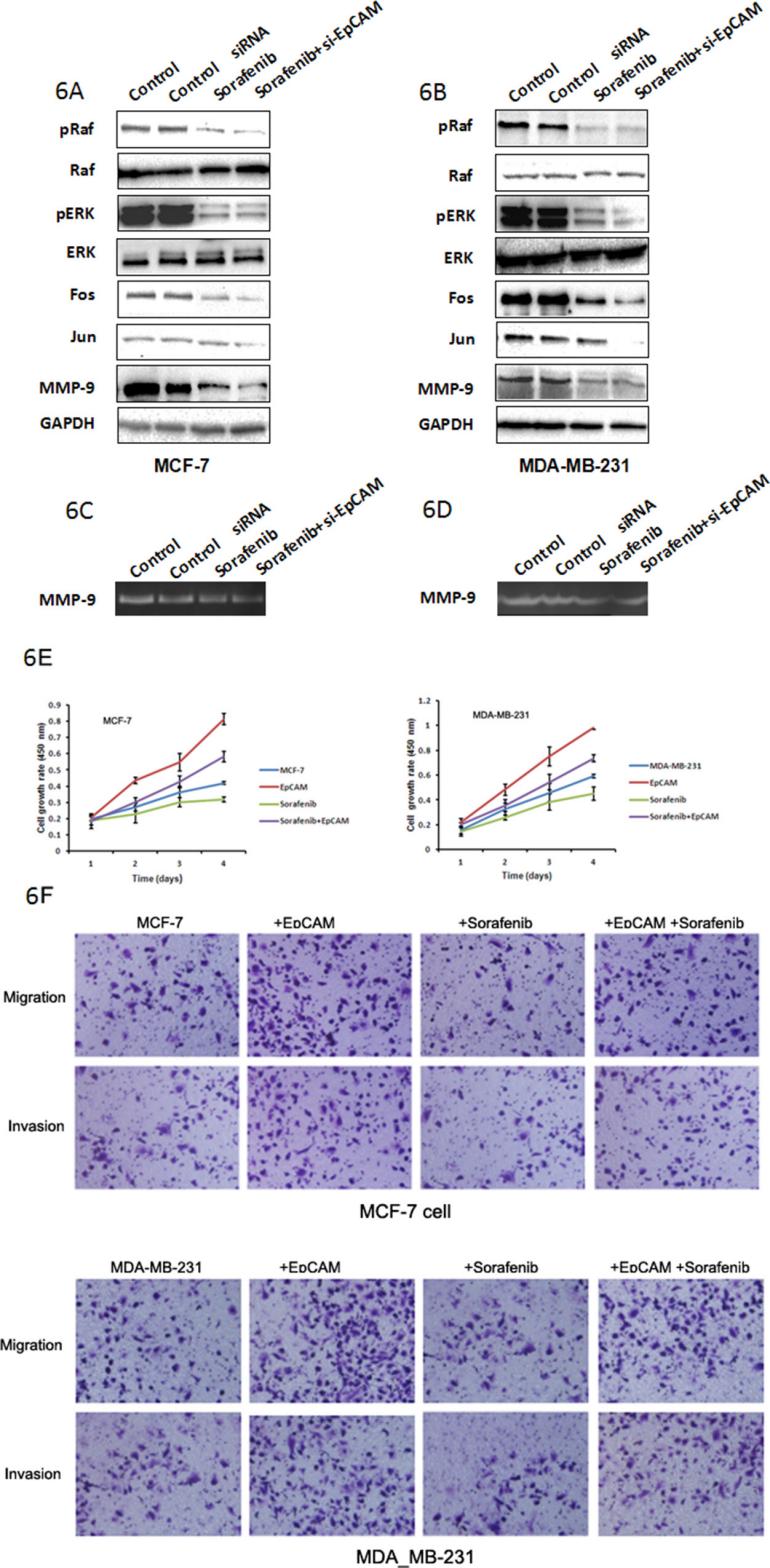
Raf are probably the downstream targets of EpCAM-mediated signaling that regulate MMP-9 expression Effect of Raf inhibitor (sorafenib) was used to analyze the effect of EpCAM on the Ras/Raf/ERK signaling pathway. MCF-7 **A.** and MDA-MB-231 **B.** cells were transfected with si-EpCAM or control siRNA, and then left untreated or were treated with 5 μM sorafenib for 48 h. Total proteins were subjected to Western blot analysis using phosphorylated Raf and ERK, Fos, Jun, and MMP-9 antibodies as described in the Materials and Methods section. MMP9 activities were determined using the gelatin-based zymography assay **C, D.** **E.** Growth curves rates were determined with a CCK-8 proliferation assay. *P < 0.01. **F.** Transwell cell migration and invasion assays affected by sorafenib and EpCAM were used to compare cell migration and invasion in the MCF-7 and MDA-MB-231 cells.

We also assessed the level and activities of MMP-9, and found that EpCAM knockdown inhibited MMP-9 expression (Fig. 5C, 5D) and activity (Fig. 5E, 5F). The expression of Fos and Jun were decreased after knockdown of EpCAM (Fig. 5C, 5D). Next, we examined the potential involvement of Ras/Raf/ERK signaling using a specific Raf inhibitor (sorafenib). Treatment of MCF-7 and MDA-MB-231 cells with 5 μM sorafenib reduced the cellular levels of pRaf, pERK, Fos, Jun, and MMP-9 (Fig. 6A, 6B), and was accompanied by reduced MMP-9 activity (Fig. 6C, 6D). We also investigated the effect of sorafenib on the overexpressed EpCAM-mediated cell biological behaviors. As shown in the Fig. 6E, EpCAM overexpression significantly enhanced the growth of MCF-7 and MDA-MB-231 cells. Raf inhibitor (sorafenib) inhibited this process and weakened the effect of EpCAM overexpression on the cell growth. Transwell assay showed that sorafenib could decrease the effect of EpCAM overexpression on the cell migration and invasion in the MCF-7 and MDA-MB-231 cells (Fig. 6F). Collectively, our studies demonstrated that MMP-9 may be an effector of breast cancer progression and metastasis mediated by EpCAM.

**Figure 7:**
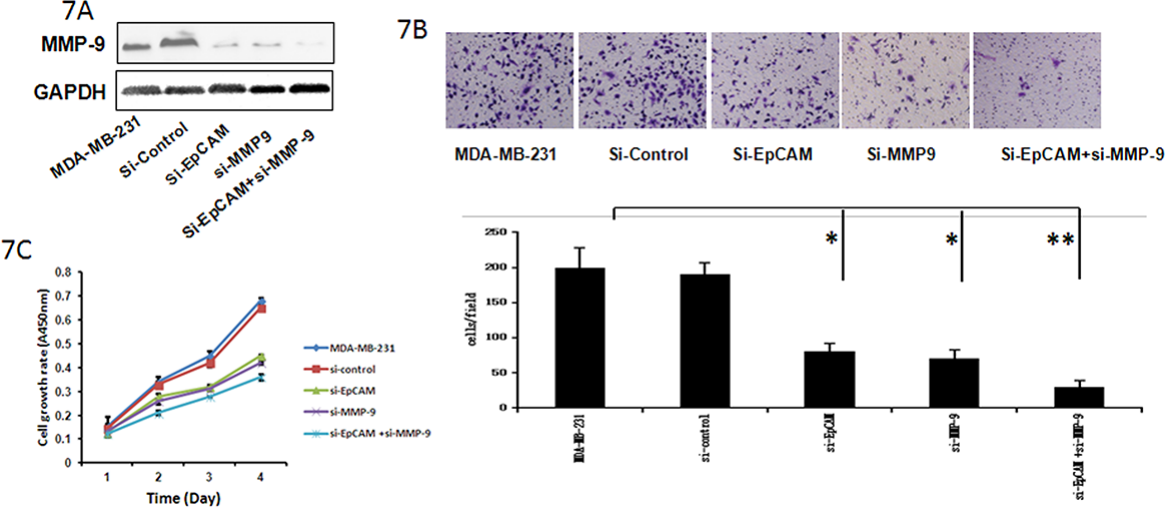
Si-EpCAM inhibited breast cancer cell growth and invasion through regulation of MMP-9 **A.** Cells were transfected with si-EpCAM, control siRNA, or and si-MMP-9 for 48 h. Cell lysates were prepared and subjected to immunoblot analysis for MMP-9. GAPDH served as an internal control. **B.** Transwell cell migration and invasion assays were used to compare cell migration and invasion after tranfection with si-EpCAM, control siRNA, or si-MMP-9. The results were expressed as mean ± SD of three independent experiments. *P < 0.05, **P < 0.01. The summary graphs are presented for the experiment. **C.** The cell growth rates were determined with a CCK-8 proliferation assay. *P < 0.05, **P < 0.01.

In addition, we investigated whether the Ras/Raf/ERK signaling pathway and MMP-9 played crucial roles in the promotion of growth and invasiveness of breast cancer cells mediated by EpCAM. Downregulation of MMP-9 by siRNA in MDA-MB-231 cells led to a significant decrease in the number of invasive cells (Fig. 7A, 7B). Furthermore, the downregulation of MMP-9 in MDA-MB-231 cells was sufficient to abolish the increase in cell proliferation (Fig. 7C). Taken together, it appears that EpCAM promotes breast cancer growth and invasion through regulation of MMP-9 expression.

### Knockdown of EpCAM inhibits breast cancer tumor metastasis *in vivo*

**Figure 8:**
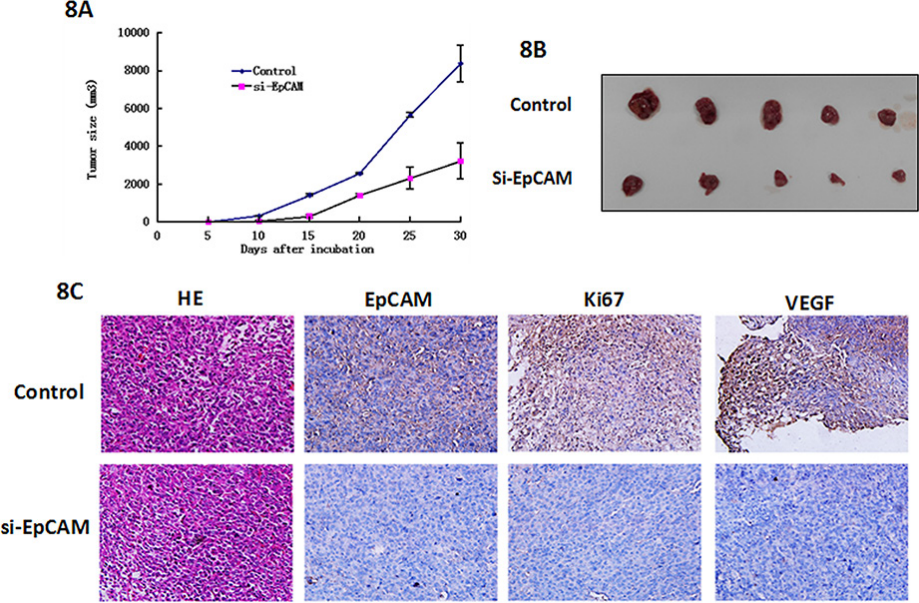
Effects of EpCAM downregulation on tumor growth and metastasis in a xenograft nude mice model **A.** Growth curves of mammary tumors after injection of si-EpCAM-transfected and control MDA-MB-231 cells into mice. The error bars represent the means ± SD (*n* = 6). **B.** *In vivo* tumor growth in the si-EpCAM group was significantly inhibited compared to the control group. **C.** Representative examples of H&E, EpCAM, Ki67, and VEGF immunohistochemistry from si-EpCAM-transfected and control cell-derived MDA-MB-231 xenografts.

To further explore the effect of EpCAM on tumor growth and metastasis *in vivo*, we injected si-EpCMA-transfected MDA-MB-231 cells into nude mice. Mice that had been injected with control MDA-MB-231 cells formed tumors on the 6^th^ day, whereas mice that had been injected with si-EpCAM-transfected cells did not form tumors until the 11^th^ day. The tumors in the si-EpCAM group grew slowly compared to those in the control group. As shown in Fig. 8A and 8B, the tumor sizes of control MDA-MB-231 cells were 8389 ± 968 mm^3^, which were significantly larger than the tumor sizes of xenografts derived from si-EpCAM-transfected MDA-MB-231 cells (3240 ± 846 mm^3^, *P* < 0.01). After 30 days, the mice were killed and tumor tissues were removed and embedded in paraffin. Tumor tissue sections were prepared, and immunohistochemistry was performed with EpCAM, Ki67, and VEGFA antibodies. The results showed the expression of EpCAM, Ki67, and VEGFA in the control group was higher than that in the si-EpCAM treatment group. VEGFA is the marker of blood vessel formation [22]. VEGFA expression decreased indicating that EpCAM plays a role in blood vessel formation. These data demonstrate that knockdown of EpCAM possesses a strong tumor suppressive role.

## DISCUSSION

EpCAM is a cell surface glycoprotein of approximately 40 kDa that is highly expressed in epithelial cancers and at lower levels in normal simple epithelia [23, 24]. EpCAM is highly expressed in rapidly proliferating carcinomas and plays an important role in the prevention of cell-cell adhesion, cell signaling, migration, proliferation, and differentiation [25, 26]. EpCAM is commonly expressed in breast cancer, and EpCAM expression in primary breast cancers is associated with a poor prognosis [27]. Despite the decline in breast cancer mortality, a number of breast cancer patients develop metastatic tumors after surgical operation. Metastasis remains the main obstacle to the effective treatment of breast cancer. In the present study, we identified EpCAM as a candidate target gene for the inhibition of breast cancer growth and metastasis.

During metastatic dissemination, cells from the primary tumor follow an orderly sequence of steps to acquire the properties required for successful metastasis. The ability to migrate and invade the basement membrane into the surrounding tissues, blood, and lymphatic vessels is one of the essential hallmarks of cancer, and is a prerequisite for local tumor progression and metastatic spread [28, 29]. Our results showed that the level of positive EpCAM staining increased with tumor stage.

Our results showed that EpCAM regulates breast cancer cell migration and invasion properties in cell culture. Our *in vitro* assay indicated that the ability of EpCAM to promote metastasis is mainly due to its effect on the regulation of breast cancer cell migration and invasion. The essential requirement for EpCAM in these processes highlights the potential for using EpCAM as a target for blocking tumor invasion in the local environment.

Our studies also point to mechanisms by which EpCAM modulates migration and invasion. We have shown that EpCAM silencing results in the decrease of MMP-9 expression. Therefore, we propose that EpCAM is required for cell migration and cancer invasion and metastasis and required controlled degradation of the extracellular matrix (ECM). However, further studies are needed to address the mechanism underlying the regulation of MMP-9 by EpCAM. MMPs are upregulated in cancer cells and play a critical role in cancer invasion and metastasis [30, 31]. MMP-mediated ECM degradation also leads to these processes [32]. MMP-9 enhances cancer progression by regulating angiogenesis, migration, proliferation, and invasion. In order to understand the connection between EpCAM and breast cancer cell invasion/migration in more detail, it will be of great interest to identify signaling cascades by which EpCAM regulates these activities. The Ras/Raf/ERK pathway has different effects on growth, apoptosis, and cell cycle arrest [33, 34].

**Figure 9:**
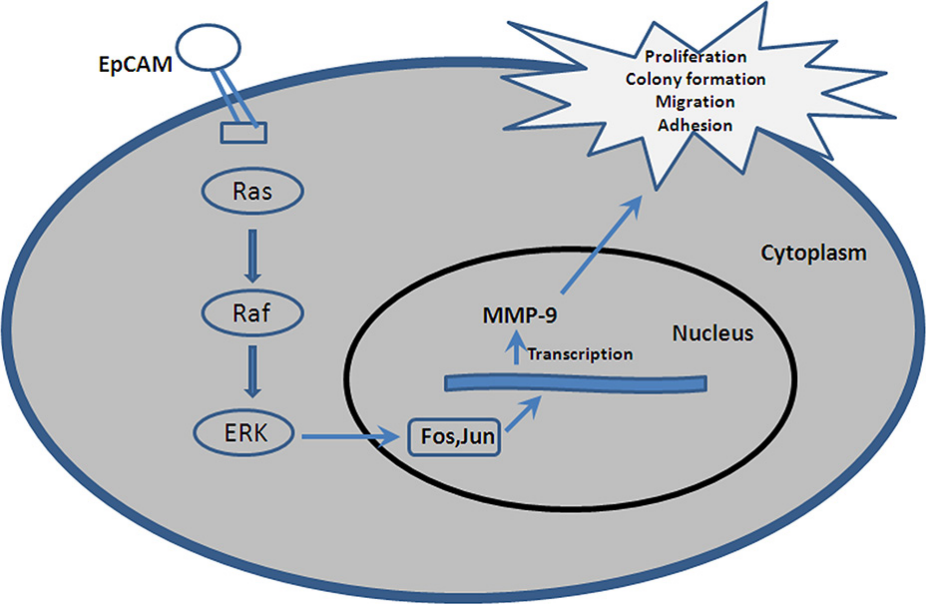
A hypothetical schematic of the contribution of EpCAM to breast cancer cells via the Ras/Raf/ERK signaling pathway The physiological function of EpCAM is to promote activation of the Ras/Raf/ERK signaling pathway, along with increased MMP-9 expression and activity.

The Ras/Raf/ERK pathway is activated by diverse mechanisms. In our study, we found that knockdown of EpCAM in breast cancer cells significantly decreased the expression of Ras, pRaf, and pERK. These data suggest that knockdown of EpCAM might affect the expression of MMP-9 through the Ras/Raf/ERK pathway. We demonstrated that inhibition this pathway could explain the effects of EpCAM silencing on migration and invasion. Yoshida and colleagues [32] found that EpCAM regulates the MAPK signaling pathway, which is essential for the response to growth factors stimulated by EGF. Consistent with their report, our study indicated that the upregulation and activation of MMP-9 via the Ras/Raf/ERK signaling pathway was required for EpCAM-mediated growth and invasion of breast cancer cells (Fig. 9). When the Ras/Raf/ERK pathway was blocked, MMP-9 expression was decreased, accompanied by attenuated proliferative and invasive capability of breast cancer cells.

Together, our current observations point to a potential oncogenic role for EpCAM in breast cancer cell invasion and migration, possibly via activation of the Ras/Raf/EFK pathway by MMP-9 upregulation through Fos and Jun. This novel mechanism of action for EpCAM is outlined in Fig. 9. Thus, EpCAM may be a potential therapeutic target for blocking breast cancer invasion.

## MATERIALS AND METHODS

### Cell culture

Breast cancer cells MCF-7 and MDA-MB-231were purchased from ATCC (USA). MCF-7 cells were maintained in medium MEM supplemented with 10% calf serum, 1% Pen/Strep, 1 mM sodium pyruvate, 1.5 g/L sodium bicarbonate, and 10 mM HEPES. MDA-MB-231 cells were cultured in L-15 containing 10% calf serum, 1% Pen/Strep and 10 mM HEPES. All cells were incubated in a 5% CO_2_ humidified atmosphere at 37°C.

### siRNA knockdown analysis

RNAi-mediated knockdown was performed with the following short interfering RNA (siRNA): EpCAM-1: 5′-UGCUCUGAGCGAGUGAGAATT-3′; EpCAM-2: 5′-UUCUCACUCGCUCAGAGCATT-3′, Negative control siRNA was used in each experiment as a non-silencing control siRNA (siRNA). All siRNAs (20 nM) targeting EpCAM was introduced in cells using lipofectamin 2000 reagent according to the manufacturer's protocol.

### Western blot

To prepare whole cell extracts, cells at 90% confluent were washed in phosphate-buffered saline (PBS) before incubation with lysis buffer (1% Triton X-100, 150 mM NaCl, 10 mM Tris, pH 7.4, 1 mM EDTA, 1 mM EGTA, pH 8.0, 0.2 mM Na_3_VO_4_, 0.2 mM phenylmethylsulfonyl fluoride, 0.5% Nonidet P-40) on ice for 10 min. The cell lysates were clarified by centrifugation at 9000 × g for 10 min and the supernatants were collected. Protein concentration was determined with the Coomassie Protein Assay Reagent using bovine serum albumin (BSA) as a standard. Cell lysates (50 μg) were separated by 10% SDS-PAGE min-gel. Samples were transferred electrophoretically to nitrocellulose membranes, blocked with TTBS containing 5% fat-free dry milk for 2 h and incubated for 3 h with the appropriate primary antibodies at the dilutions recommended by the suppliers. After incubation with a HRP-conjugated anti-goat secondary antibody, immunoreactive proteins were visualized with ECL detection system. Western blots shown are representative of at least three independent experiments. Densitometry of each band for the target protein was quantified by densitometry analysis with Labworks 4.6. The protein band intensity was quantified by the mean ± SEM of three experiments for each group as determined from densitometry relative to β-actin.

### Cell growth and foci formation assays

To perform growth assay, cells were seeded at a density of 1000 cells per well in 96-well plate. The cell growth rate was detected using Cell Counting Kit-8 (CCK8, Dojindo Molecular Technologies) according to the manufacturer's instruction. Triplicate independent experiments were carried out.

For foci formation assay, 1000 cells were seeded in each well of a six-well plate. After 6 days of culture, surviving colonies were counted with crystal violet staining. Triplicate independent experiments were carried out.

### Transwell cell migration and invasion assays

The transwell cell migration assay and invasion assay were performed using BD chambers containing polyethylene terephthalate membranes of 8 μm pore size and BD Matrigel Invasion Chambers with 8 μm porosity (BD Biosciences, Bedford, MA) according to the manufacturer's instructions, respectively. Cells were suspended in serum-free DMEM/F12 at a density of 8.0 × 10^4^ cells per well onto the upper chambers and DMEM/F12 with 10% fetal bovine serum was added to the lower chambers. Cells that had migrated through to the bottom of the insert membrane were fixed, stained and counted from six random fields under *a* × 20 objective lens. The experiments were repeated three times.

### Gelatin zymography assay

MMP-9 activity was detected using the gelatin zymography assay. Cells (2 × 10^5^ cells/ml) were seeded into six-well plates and treated. The supernatants were collected and used as the samples. Cells were also collected, and proteins were extracted using lysis buffer (50mM Tris-HCl, 150mM NaCl, 0.02% NaN3 and 1% NP-40) for 30 min. The protein concentration was determined using the Bradford assay. A concentration of 40 mg of total proteins of supernatants were loaded per lane and electrophoresed on 8% SDS-polyacrylamide gels copolymerized with 1% gelatin. After electrophoresis, the gels were washed five times in 2.5% Triton X-100 (20 min each) and two times in buffer without Triton X-100 to remove Triton X-100, and then incubated in 50 mmol/l Tris-Cl, pH 7.6, and 5 mmol/l CaCl_2_ (18 h, 37 1C). The gels were stained with 0.1% Coomassie blue R250 and destained in 10% isopropanol and 10% acetic acid in H_2_O. MMP-9 was detected as transparent bands on the blue background of a Coomassie blue-stained gel.

### Quantitative reverse-transcription PCR (q PCR)

RNA was extracted using TRIzol reagent, according to the manufacturer's recommended protocol (Invitrogen). qRT-PCR was performed using SYBR^®^ Premix Ex Taq™ (TAKARA, Japan)according to the manufacturer's instructions. The primer sequences used were as follows: EpCAM forward primer 5′-TGCTGTTATTGTGGTTGTGGTG-3′, reverse primer 5′- TACTTTGCCATTCTCTTCTTTCTGG-3′; GAPDH forward primer 5′- GCACCGTCAAGGCTGAGAAC-3′, reverse primer 5′-TGGTGA AGACGCCAGTGG-3′; GAPDH was used as a loading control. The experiments were repeated a minimum of three times to confirm the results.

### Immunofluorescence staining

Immunofluorescence staining was performed on cultures after fixing the cells in 4% paraformaldehyde for 15 min. Non-specific binding was blocked with a 5% BSA-phosphate buffer solution for 1 hr. The cells were then incubated with rabbit polyclonal antibodies specific for Ki67 diluted at 1: 100 in blocking buffer overnight at 4°C. After washing with PBS, the cells were incubated with secondary antibodies diluted at 1: 200 in blocking buffer for 2 hr at room temperature. The cells were washed with PBS and incubated with DAPI for 10 min at room temperature. Images were obtained on a fluorescence microscope.

### *In vivo* assays for tumour growth and metastasis

The *in vivo* tumorigenesis and metastasis assays were performed, as followed. Si EpCAM and si RNA control transfected MDA-MB-231cells (2 × 10^6^ ) were injected into the left and right dorsal flank of 5-week-old female nude nice, respectively.

Growth curves were plotted, based on mean tumor volume at each time point, for each experimental group. The tumor dimensions were measured every 3 days using a digital caliper. The tumor volume (mm^3^) was calculated as follows: V = ab^2^/2, where a and b are the largest and smallest tumor diameters measured at necropsy, respectively. After 6 weeks, the mice were killed and the tumor tissues were harvested for use in further experiments.

### Tissue microarray (TMA) and immunohistochemistry (IHC) analysis

Breast cancer and normal tissue microarray (TMA) sections with stage and grade information were bought from US Biomax, Inc. (Rockville, MD). For IHC analysis, the TMA sections were deparaffinized in 100% xylene and rehydrated in graded ethanol solutions. The sections were then boiled under pressure in citrate buffer (pH 6.0) for 5 min for antigen retrieval. TMA sections were incubated for 1 h with a primary antibody in TBS containing 1% bovine serum albumin. After washing, these sections were incubated with anti-rabbit horseradish peroxidase-conjugated antibody. Images were taken with an Aperio scanscope CS system (Vista, CA). TMA sections were semiquantitatively analyzed by two other investigators.

### Statistical analysis

The quantitative data derived from three independent experiments are expressed as means (±SD). Unpaired Student's *t*-tests were used to analyze between group differences that is repeated and *p* < 0.05 was considered statistically significant.
